# A novel smartphone application for the tracking of procedural numbers and trainee experience in gastrointestinal endoscopy

**DOI:** 10.1186/s12911-023-02145-z

**Published:** 2023-03-31

**Authors:** Sergio Zepeda-Gómez, Andrea Dávila-Cervantes, Aldo J. Montaño-Loza, David Yang, Daniel C. Baumgart, Karen Kroeker, Brendan P. Halloran

**Affiliations:** Division of Gastroenterology, University of Alberta, Zeidler Ledcor Centre, Edmonton, Canada

**Keywords:** Gastrointestinal Endoscopy, Endoscopy training, Quality indicators endoscopy

## Abstract

**Objectives:**

The tracking and documentation of procedures in gastrointestinal endoscopy including therapeutic interventions is an essential but challenging process. The University of Alberta has developed a smartphone app to help facilitate this task. This study evaluated the functionality, usefulness, and user satisfaction of this app.

**Methods:**

Four Gastroenterology (GI) residents and two therapeutic endoscopy fellows participated in the study. The trainees submitted all their data into the app from the procedures in which they participated hands-on for one year, data was collected and analyzed on the app and the website associated with it.

**Results:**

Trainees were able to register the procedures immediately after each procedure without difficulty, this data was available to be reviewed at anytime in the app and associated website. Furthermore, the data collected was able to be transformed into tables and graphs on the app website.

The total number of procedures and therapeutic interventions performed were easily accessed in the app and website at anytime. The app facilitated the calculation of the cecal intubation rate in colonoscopy and the cannulation rate in ERCP for the therapeutic endoscopy trainee. Trainees reported excellent experience with the app capabilities.

**Conclusions:**

A novel smartphone app was useful in collecting meaningful data submitted by gastrointestinal endoscopy trainees, furthermore, through an associated website, it was capable to create graphs and tables to show and facilitate the calculation of meaningful data such as key performance indicators.

**Supplementary Information:**

The online version contains supplementary material available at 10.1186/s12911-023-02145-z.

## Introduction

The number, type and quality of endoscopic procedures performed is an important part of Gastroenterology and Surgery training Programs. These parameters may reflect the acquisition of endoscopic skills and competency. Some examples of current metrics for endoscopic quality include cecal intubation rate in colonoscopy or, for advanced trainees, cannulation rate in endoscopic retrograde cholangiopancreatography (ERCP). Documenting all endoscopic procedures—including therapeutic interventions and milestones—throughout the training period can be challenging and time-consuming but is essential in understanding trainee ability and progression. There is a need for a tool that records trainee experience in an effective and simple way.

Here we describe a novel smartphone software application (app) named Endostation developed and tested by the Division of Gastroenterology at the University of Alberta. This app was designed to facilitate fast procedural documentation and to track meaningful data of trainees in gastrointestinal endoscopy.

The purpose of this pilot study was to evaluate the functionality, feasibility and usefulness of a smartphone application developed for documenting and analyzing the number of endoscopic procedures and therapeutic interventions performed by endoscopy trainees over time.

## Methods

Four Gastroenterology (GI) residents in their first year of training and two therapeutic endoscopy fellows agreed to participate in the study using the smartphone app to document the procedures that they performed. The participants (trainees) gave their informed consent to participate in the study. We encouraged the trainees to submit this data immediately after each procedure. The participants were instructed to register all the procedures they started (*hands-on*) and document the therapeutic interventions that they performed independently (without *hands-on* help from the supervisors) If there was minimal (not meaningful) hands-on intervention from supervisors, these procedures could be registered as well. Trainees were instructed to report if there were any issues with the functionality of the app when submitting their data. The objective of the app was to document and calculate the number of procedures performed and monitor the type of interventions the trainees independently achieved during a one-year training period. This documentation included the number of procedures and their characteristics as well as therapeutic interventions performed by the trainees. This data was subsequently analyzed by dividing the twelve months of training into tertiles (4-month periods) to evaluate if it could be useful to determine progress in some key performance indicators.

At the end of the trial period trainees were asked to fill out a satisfaction survey regarding the experience with the app.

### Description of the smartphone app

**Fig. 1:**
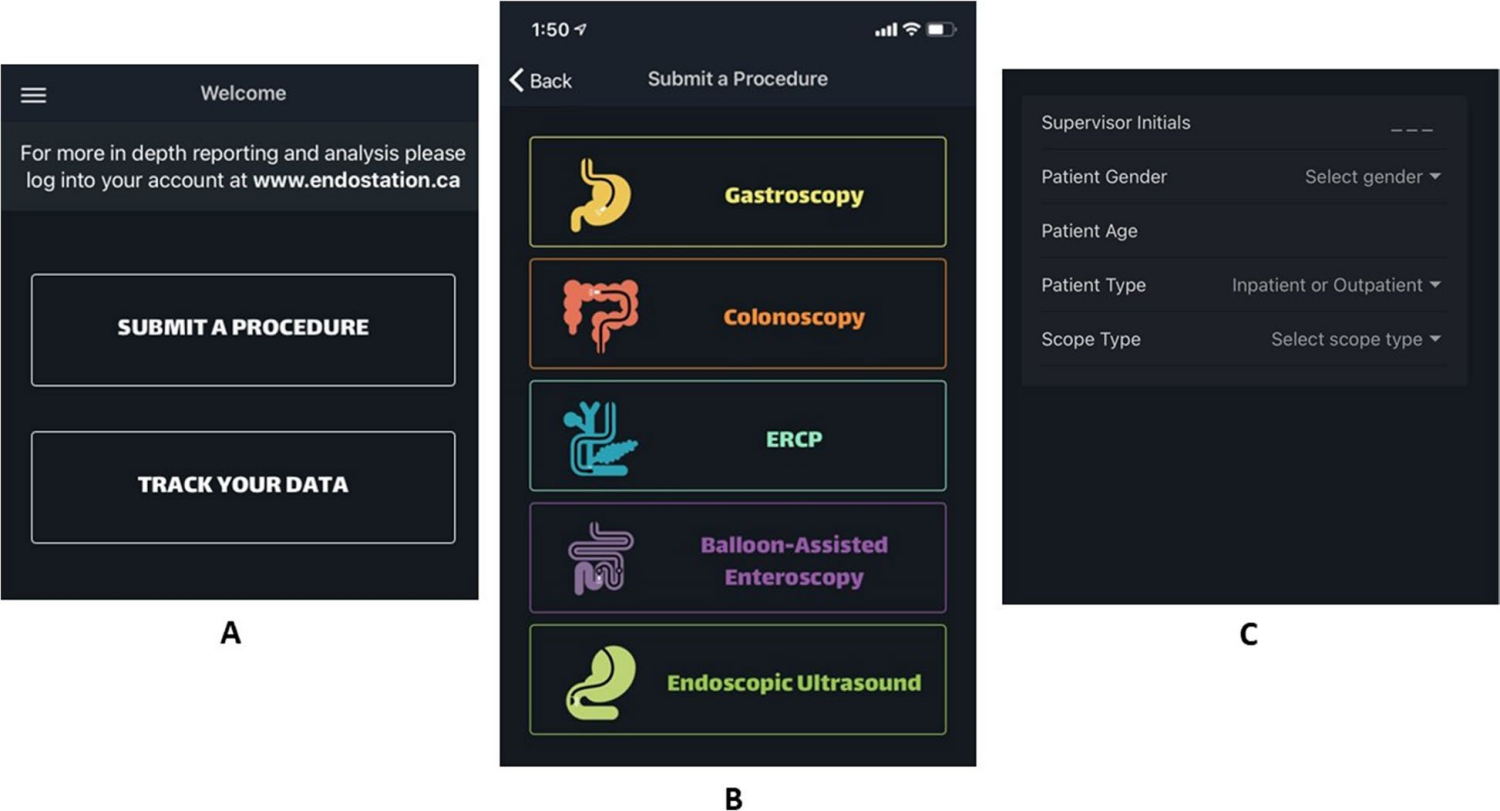
Screenshot of the app menu of the different procedures included for data tracking in app

**Fig. 2:**
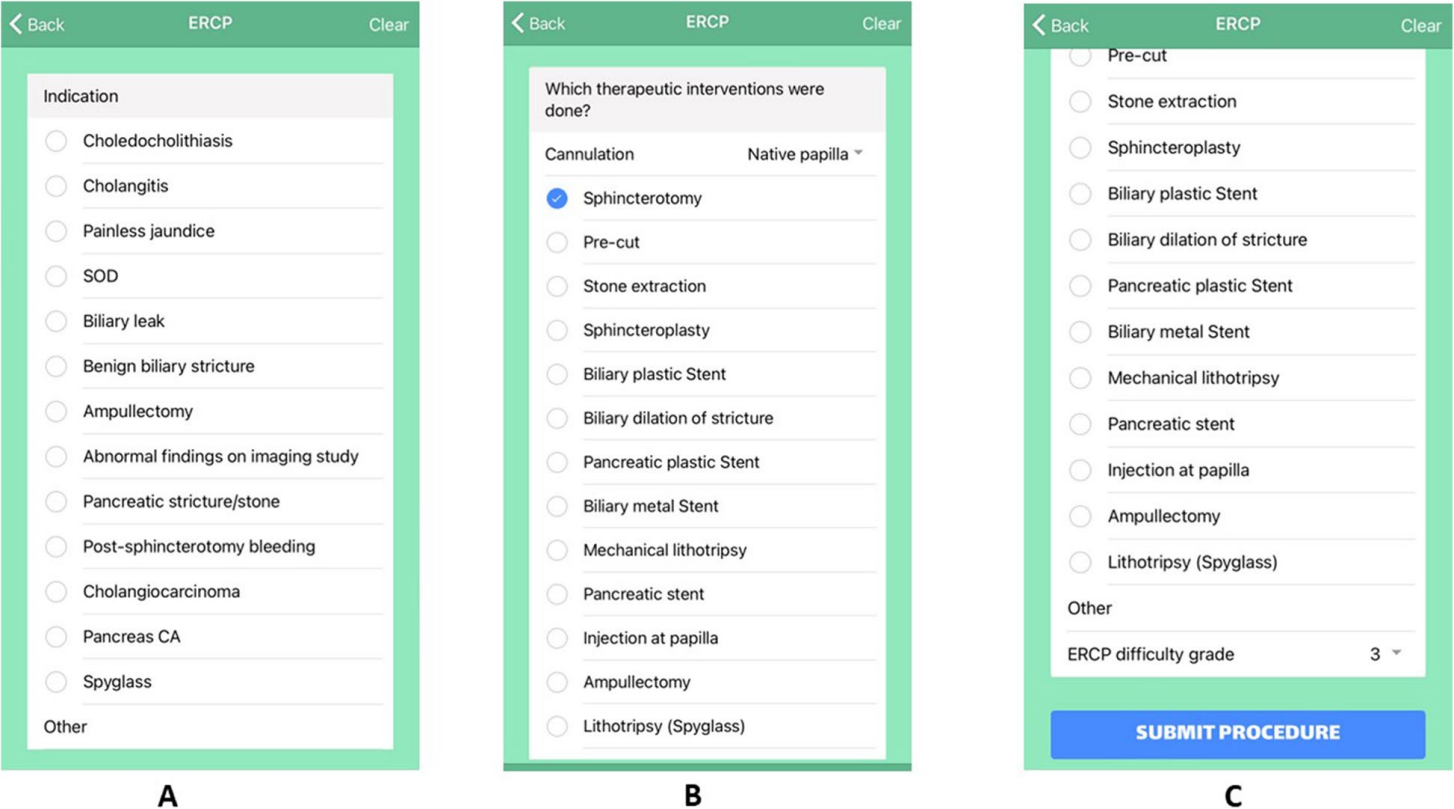
Screenshot of the app menu of ERCP indications and therapeutic interventions

Endostation (University of Alberta. EndoStation Mobile App—Skills Tracker) is a smartphone app conceived and developed by members of the University of Alberta Division of Gastroenterology (SZG, BH) in collaboration with Digital Tea Web Design (Edmonton) to facilitate documentation and tracking of procedures performed by endoscopy trainees. Every user must create their own account to be able to register data from 5 endoscopic procedures: Gastroscopy, Colonoscopy, ERCP, Balloon-assisted enteroscopy (BAE) and Endoscopic Ultrasound (EUS). Beyond procedure type and date, data collection also includes the initials of supervising faculty, patient age and gender, inpatient or outpatient status and the type of endoscope used (Fig. 1). After this, trainees must select the indication of the procedure and whether the procedure was diagnostic (no endoscopic intervention performed) or therapeutic (trainee performed an endoscopic intervention). A dropdown menu shows the lists of indications and therapeutic interventions that can be selected for all procedures (Additional file 1: Appendix 1, 2). More than one option can be chosen from each list, Fig. 2 shows a screenshot of the options for ERCP. Additional detail is captured per procedure type: for example, the segment of colon reached during colonoscopy (sigmoid, splenic flexure, hepatic flexure, cecum). For ERCP, the trainee may choose from 4 different options regarding cannulation of the papilla: *native papilla*, *prior sphincterotomy*, *failed* (cannulation was attempted but not achieved) or *did not try (the trainee did not attempt cannulation)* and the grade of difficulty based on the ASGE grading system (Additional file 1: Appendix 3) [1]. No confidential patient data can be documented in the app. The user is encouraged to submit the data immediately after finishing each endoscopic procedure since the app syncs to the smartphone calendar to document the date of every procedure accurately. Registering the procedure data takes approximately 30 s or less, including cases involving therapeutic interventions. Trainees must be aware that they should only register procedures in which they have hands-on involvement from the start. In the case of colonoscopy, they must document the furthest landmark reached without any hands-on assessment by the supervisor, this allows for subsequent accurate calculation of cecal intubation rate. Trainees can also document if they found colonic polyps (for subsequent calculation of polyp detection rate). Furthermore, they should only document therapeutic interventions that they performed by themselves. To improve the accuracy of the data, trainees also register the initials of their staff physician supervisor for each procedure. We provide trainees with a list of staff initials to ensure this data is consistent. The app is linked to a website (https://endostation.ca) where the submitted data can be accessed by the user and presented in various ways—including graphs that show the total number of procedures, procedure indication and therapeutic interventions performed. All data and graphs can be downloaded and shared with supervisors or the Program Director at any time. This feature facilitates the monitoring of a trainee’s individual experience and skill progression based on achieved quality metrics. In theory, this may help to evaluate the need for changes or interventions in the Program at any time during their course of training. Endostation is available for download and runs on iOS and Android platforms. This study was approved by the Ethics Research Committee at the University of Alberta (Pro00078847).

### Primary objective

To evaluate the functionality, feasibility, and usefulness of a newly developed smartphone app for documenting of the number of endoscopic procedures and therapeutic interventions performed over time by GI and therapeutic endoscopy trainees.

### Secondary objectives

To evaluate the capability of the app to facilitate the calculation of meaningful data including some key performance indicators (KPIs) over time based on the number of successful milestones performed independently by the GI residents and therapeutic endoscopy fellows. To evaluate the satisfaction level of the trainees using the app.

### Statistical analysis

Continuous variables are described as mean and standard deviation (SD) and the *Mann*–*Whitney U test* was used to compare differences in means. For categorical variables, descriptive statistics are presented as absolute numbers and percentages and the Pearson chi-square or Fisher’s exact test were used where indicated to determine associations between categorical variables.

Statistical analyses were performed using SPSS (SPSS for Windows, version 26.0, SPSS, Chicago, IL) and a *p*-value < 0.05 was used to determine statistical significance.

## Results

**Fig. 3:**
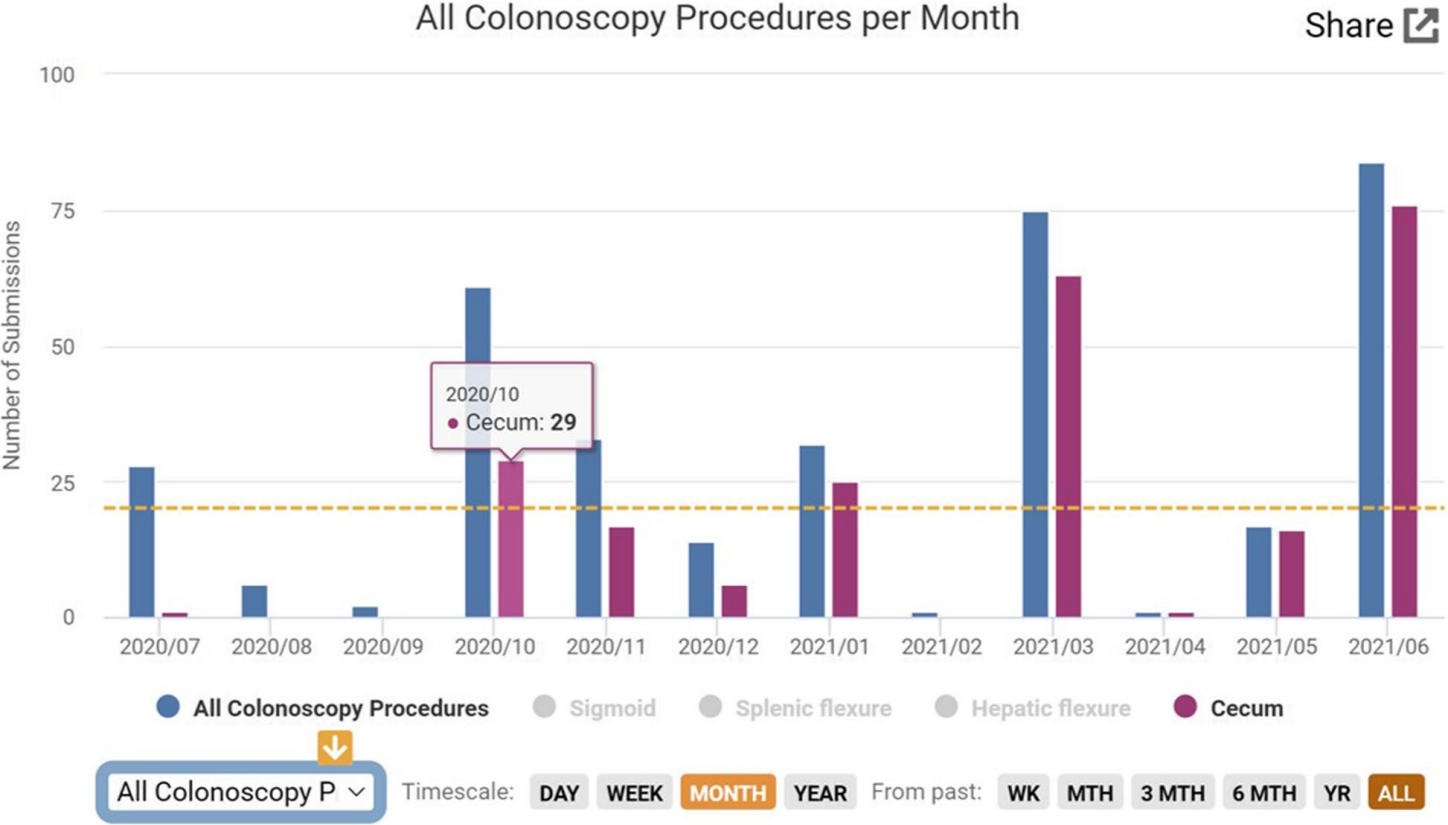
Screenshot of a graph created on app website showing the number of total colonoscopies per month (blue bar) for a GI trainee along with the procedures where cecal intubation was achieved (red bar). Notice that the exact number is displayed when the cursor is placed over the respective bar

Data submitted by four GI residents and by two advanced endoscopy fellows in a 12-month period was successfully collected and evaluated. All the data submitted by the trainees was able to be accessed in the app and converted into graphs on the associated website at any point during the training period. There were no major issues reported by the trainees in regard to the functionality of the app to register and submit the data after each procedure. We present here the numbers of the data analysis in two GI residents (trainees 1 and 2) and one advanced endoscopy fellow. The information from the app and website showed that the GI trainee 1 had a higher number of gastroscopies and colonoscopies over one year than trainee 2. Esophageal dilation and cold snare polypectomy were the therapeutic interventions most reported among the GI residents (Table 1). The cecal intubation rate was significantly higher for both GI trainees when comparing the first 4-months of training with the last 4-month period: 24% vs 88% (*p* =  < 0.05), and 15% vs 42% (*p* =  < 0.05) for trainee 1 and trainee 2 respectively (Table 2). Figure 3 is a graph generated via the Endostation website displaying cases in which cecal intubation was achieved along with the total number of colonoscopies performed over time for trainee 1. For the therapeutic endoscopy trainee, the number of ERCP procedures and characteristics in the 12-month are shown in Table 3; the most common indication was choledocholithiasis and stone extraction the most frequent therapeutic intervention (with balloon and/or basket). For EUS, the most common indications were evaluation to confirm/rule out common bile duct stones as well as evaluation of pancreatic mass. The EUS numbers by month, stations completed, and therapeutic interventions are presented in Table 4.

**Table 1:** Total procedure numbers and examples of therapeutic interventions in gastroscopy and colonoscopy for two GI trainees (12-month period)

**Trainee 1**	**Jul**	**Aug**	**Sep**	**Oct**	**Nov**	**Dec**	**Jan**	**Feb**	**Mar**	**Apr**	**May**	**Jun**	**Total**
Gastroscopy	60	17	12	55	41	41	57	2	63	0	32	74	**454**
Colonoscopy	26	4	2	46	33	14	32	1	75	1	17	84	**335**
**Trainee 2**	**Jul**	**Aug**	**Sept**	**Oct**	**Nov**	**Dec**	**Jan**	**Feb**	**Mar**	**Apr**	**May**	**Jun**	**Total**
Gastroscopy	11	35	0	58	46	8	6	7	5	58	19	52	**305**
Colonoscopy	4	9	0	33	23	4	3	2	5	46	7	34	**170**
**Therapeutic interventions**					**Trainee 1** **(n)**					**Trainee 2** **(n)**			
Esophageal dilation (balloon and bougie)					24					13			
PEG-tube insertion					12					10			
Variceal banding esophagus					9					12			
NJ-tube insertion					5					1			
Gastric hemoclips					5					5			
Duodenal hemoclips					5					2			
Gastric APC					4					3			
Cold snare polypectomy					49					16			
Hot snare polypectomy					9					1			
Colonic hemoclips					6					2			
**Total**					128					65

**Table 2:** Cecal intubation rate for 2 GI trainees (4-month periods)

	**Jul-Oct** **CI/n**	**Nov-Feb** **CI/n**	**Mar-Jun** **CI/n**	**Total**
Trainee 1	19/78	48/80	156/177	223/335
Cecal Intubation rate	24%*	60%	88%*	66%
Trainee 2	7/46	13/32	39/92	59/170
Cecal Intubation rate	15%*	40%	42%*	34%

^*^p =  < 0.05 (chi-square), *n* number of colonoscopies, *CI* cecal intubation

**Table 3:** ERCP data for one advanced endoscopy trainee including therapeutic interventions (12-month period)

**Number of ERCP procedures by month**														
**Month**		1	2	3	4	5	6	7	8	9	10	11	12	Total
Trainee 1		32	59	57	67	61	44	42	54	41	38	23	28	546
Indications														
Choledocholithiasis (cholangitis)								357 (44)						
Painless jaundice/malignancy								53						
Biliary Leak								35						
Benign biliary stricture								47						
Pancreatic stricture/stone								25						
Ampullectomy								6						
SOD								4						
Post-sphincterotomy bleed								8						
Spyglass								36						
**Therapeutic Interventions**														
Sphincterotomy								234						
Stone extraction (basket/balloon)								314						
Plastic stent placement								85						
Metal stent placement								64						
Biliary stricture dilation								17						
Pancreatic stent								9						
Sphincteroplasty major papilla								53						
Pre-cut								5						
**Total**								781						
**ERCP difficulty Grade**														
**1**								226						
**2**								268						
**3**								44						
**4**								8						
**Total**								546

**Table 4:** EUS data for one advanced endoscopy trainee (12-month period)

Number of EUS procedures by month												
**Jul**	**Aug**	**Sep**	**Oct**	**Nov**	**Dec**	**Jan**	**Feb**	**Mar**	**Apr**	**May**	**Jun**	**Total**
12	28	25	48	42	47	28	60	55	33	37	22	437
**Indication**						**Total**						
Rule out Choledocholithiasis						184						
Evaluation ± biopsy of pancreatic mass						183						
Celiac plexus block (Abdominal pain)						8						
Evaluation gastric submucosal mass						22						
Evaluation duodenal submucosal mass						13						
Evaluation esophageal submucosal mass						5						
Staging ampullary mass						12						
Rectal EUS						10						
**Stations completed**												
Station 1 (trans-gastric)						416						
Station 2 (duodenal bulb)						393						
Station 3 (ampullary)						374						
**Therapeutic Intervention**												
Celiac plexus neurolysis						4						
Fine-needle biopsy pancreas mass						42						
Fine-needle biopsy pancreas cyst						9						
Fine-needle biopsy submucosal mass						11						
Fine-needle biopsy lymph node						9						

**Fig. 4:**
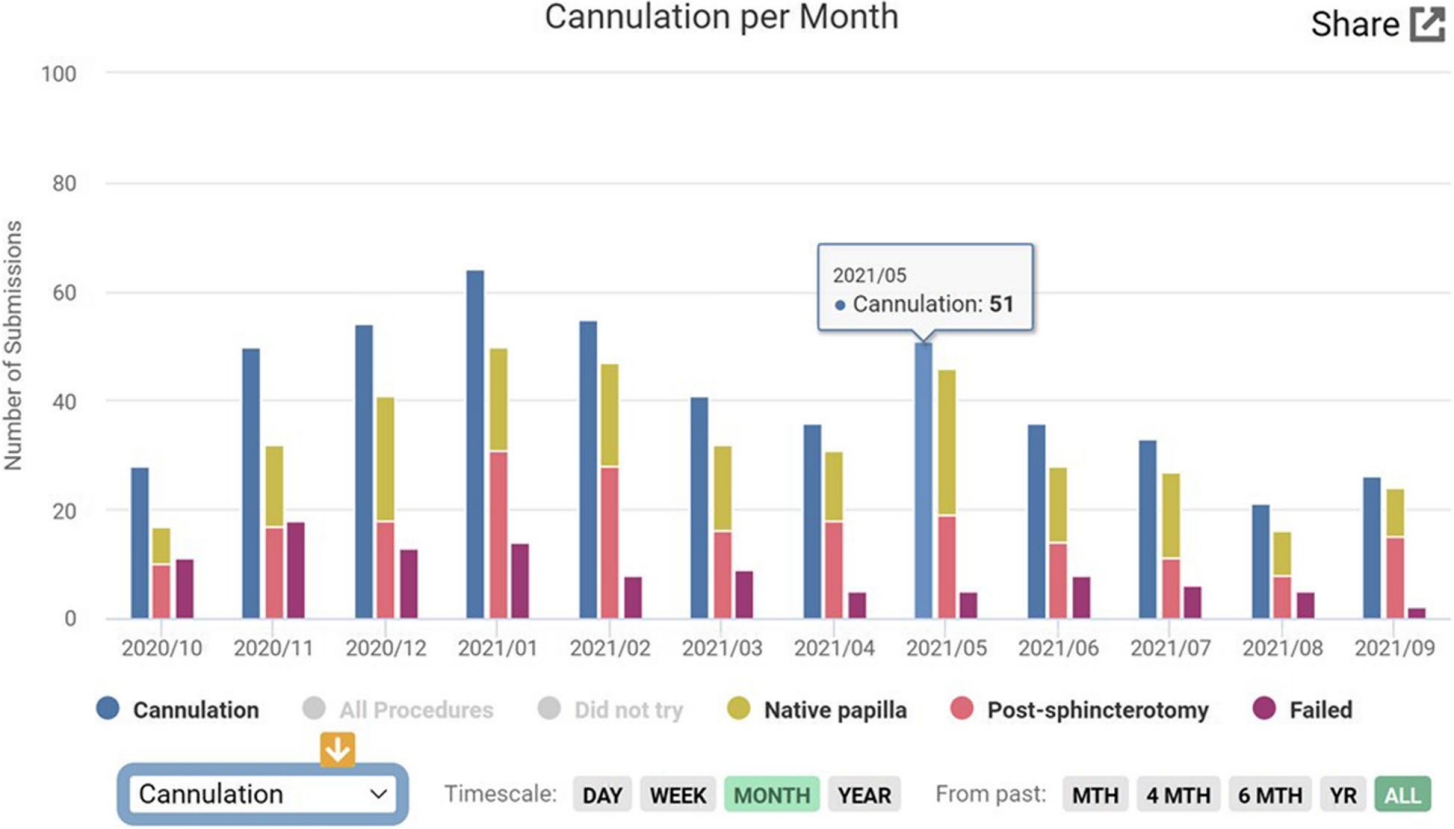
Screenshot of successful number of cannulations over time (12 months). This graph includes the cases with previous sphincterotomy (pink bar) and native papilla cases (yellow bar), the sum of both represents the total number of successful cannulations compared with the total number of cannulation attempts (blue column). The burgundy bar reflects the number of failed cannulations. Notice that there is also an option to display the number of total procedures and the procedures where cannulation was not attempted by the trainee (did not try)

The cannulation rate in ERCP for the last 4-month period of training was significantly higher when compared with the first 4-months (85% vs 71% respectively, *p* =  < 0.05). These included native papilla and post-sphincterotomy cases. When analyzing only native papilla cannulation rates, even when the cannulation rate decreased, there was a statistically significant difference between the first 4 months of training when compared with the last 4-month period (53% vs 75%, *p* =  < 0.05) (Table 5). Figure 4 shows a graph created at the app website depicting the successful number of cannulations over time (12 months). The trainees survey after the completion of the study showed that they were highly satisfied with the functionality, easiness of use and access of their data from their endoscopic procedures with an overall score of 4.8 out of 5 (Table 6).

**Table 5:** ERCP cannulation data for one advanced endoscopy trainee (divided in 4-month periods)

	**Jul-Oct**	**Nov-Feb**	**Mar-Jun**	**Total**
***Successful cannulation of common bile duct***				
Total number of cases with cannulation attempt	196	183	123	495
Successful Cannulation (including post-sphincterotomy cases)	140	156	99	391
*Cannulation rate*	71%*	85%	85%*	80%
Successful cannulation including only native papilla cases	64/120	75/102	51/68	190/290
*Cannulation rate native papilla*	53%*	73%	75%*	65%
***Failed cannulation***				
Total number of cases with cannulation attempt	196	183	123	495
Number of failed cannulations	56	27	21	104
*Failed cannulation rate*	28%*	14%	17%*	21%

*p* =  < 0.05 (chi-square)

**Table 6:** Satisfaction Survey about the overall experience with the smartphone app among 6 trainees

	**Strongly disagree**	**Disagree**	**Neutral**	**Agree**	**Strongly Agree**	**Overall**
1. The Endostation App is easy to use	1	2	3	4	5	4.8
2. The Endostation App is useful to me	1	2	3	4	5	5
3. The Endostation App is easy to learn	1	2	3	4	5	5
4. The information from the App is relevant to my work	1	2	3	4	5	5
5. Information I get from the App is accurate	1	2	3	4	5	4.8
6. The information is presented in a useful format	1	2	3	4	5	5
7. I can retrieve information I need easily	1	2	3	4	5	5
8. Overall, I am satisfied with the Endostation App	1	2	3	4	5	5
9. Using the App has helped me to log all the endoscopic procedures in a timely manner	1	2	3	4	5	4.8
10. Using the App has helped me identify the need to actively seek for specific learning opportunities	1	2	3	4	5	4.5
11. The Endostation App has facilitated the identification of trends and patterns in my training	1	2	3	4	5	4.5
**Total**						**4.8**

## Discussion

### App functionality

In this manuscript, we describe the first implementation of a novel smartphone application designed to aid in endoscopic activity reporting for trainees. We perceived a major unmet need in endoscopy training programs for a method of collecting secure and accurate data to help evaluating quality metrics. Since GI Endoscopy is constantly evolving, both in terms of technology and techniques, ensuring adequate training and competence is a challenge for Gastroenterology Programs around the world. Moreover, there has been an important shift towards patient-centered quality and competency-based training in endoscopy [2, 3]. An essential element of adequate training in GI endoscopy is enabling trainees to perform the recommended number of procedures to achieve competence. Furthermore, every Program requires preceptors responsible for demonstrating procedural techniques, evaluating skills and providing effective feedback with the ultimate goal of assessing competence according to predetermined criteria [4]. The American Society for Gastrointestinal Endoscopy (ASGE) has published guidelines for privileging, credentialing and proctoring trainees to perform GI endoscopy. These guidelines include the skills and minimum number of each procedure that should be performed before assessment of competency [5, 6].

Many training programs in Medicine have incorporated the use of personal digital assistants (PDAs) into medical education and clinical care, particularly as a learning platform where different course materials and references are shared in addition to tracking the trainees’ clinical exposure. These PDA’s, the predecessors of smartphones have been recognized as useful in physicians’ practices for rapid response to communication, error prevention, data management and accessibility [7]. Multiple studies show a wide acceptance of smartphones by health care professionals during recent years [8, 9]. Smartphones are becoming popular for clinical use among clinicians, residents and medical students [10, 11]. In a 2012 study, Mosa et al. discussed a total of 83 smartphone healthcare applications based on their varying functionalities and supported platforms. The vast majority were disease diagnosis and medical calculator applications, only one application was listed for logging surgical cases and procedures [12, 13].

The use of logbooks has been a mandatory requirement in many countries to complete and stratify specialization/registrar training in the fields of Family Medicine, Surgery and Emergency Medicine. The logging of the endoscopic procedures performed by trainees is supposed to be a mandatory requirement in GI Endoscopy and Advanced Therapeutic Endoscopy. However, the methods by which fellows track their procedural experience vary widely (handwritten, paper logs, personal cards, computer databases etc.). The lack of a systematic documentation method for logging procedures and associated interventions presents challenges in terms of practicality, data security, accuracy, and analysis. The Endostation app was developed to simplify these requirements in an effective, fast and practical way without compromising confidentiality. There were no issues during the study regarding the functionality of the app and subsequent access to data within the app itself or in the associated website.

### Usefulness of data collected

Regarding the importance of the data collected, beyond the total numbers of procedures performed, other parameters known as “competency milestones” or “key performance indicators (KPIs)” may be more reliable indicators of skills acquisition including cecal intubation rate in colonoscopy and cannulation rate in ERCP [14]. Some of these milestones are included in the data that must be submitted by trainees using Endostation. For example, in order to accurately estimate the cecal intubation rate, trainees must select the segment of colon reached for every colonoscopy in which the purpose was to advance all the way to the cecum. Other quality indicators data that the app provides are polyp detection rate (the app asks the user if there were polyps detected during colonoscopy) and major papilla cannulation in ERCP. In our study, trainee 1 in Gastroenterology achieved a high cecal intubation rate by the end of his first year of training which correlated with higher number of procedures performed as shown in Fig. 2. Regarding ERCP, trainees must demonstrate a high papillary cannulation rate at the end of their training period. The ASGE task force considers acceptable for competency a cannulation rate of > 90% in native papilla and recommends further training when this rate is below 80% [15]. The trainees are presented with different cannulation outcomes to choose from, these include “native papilla”, “post-sphincterotomy”, “failed” or “did not try”. This allows a more accurate registry of the different types of cannulation scenarios and gives the supervisor a better idea of the evolution of skills over time with regards to this important milestone. To our knowledge, there is no other data collecting system that discriminates between these types of scenarios in ERCP cannulation. The trainee must also register the ERCP grade of difficulty based on the ASGE grading system [1]. This is of particular interest as a recent study showed that technical competence was achieved for Grade 2 ERCP at 300 cases [16]. Similarly, the documentation of observed landmarks/stations in EUS is needed to be registered as this is another important performance measure. The trainees have also the option of registering the number of successful therapeutic interventions performed independently, for example, variceal band ligation, polypectomy, esophageal dilation, etc. To our knowledge, there is no consensus about the required/recommended minimum number of therapeutic interventions performed independently by GI trainees. As noted in the Gastroenterology trainee data, there is discrepancy between the numbers of procedures performed during their first year. Trainee number 1 performed a significantly higher number of procedures than trainee number 2, furthermore, trainee 2 had longer periods with minimal endoscopic training than trainee 1 (Table 1). This presentation of data has the potential to be useful when considering the ideal schedule for endoscopic exposure training, there have been different ways proposed such as endoscopy blocks vs longitudinal experience [17]. As this is a pilot study, which main objective was to evaluate the feasibility, functionality and usefulness of the app, it is important to acknowledge that at this point, Endostation is not an evaluation tool but an instrument to gather data that has the potential to assist in tracking overall experience in the training schedule, thereby aiding in the information regarding acquisition of skills by endoscopy trainees. In the future, the app could have the potential to expand its capabilities and include other quality indicators as well as a faculty assessment component that could enhance its value.

### Limitations

We were not able to evaluate the compliance of the trainees in entering all their endoscopic experience; this is one of the drawbacks of this study and a subject for future analysis. Another limitation may be the fact that trainees must be aware that they should only register the procedures in which they did not have (or had minimal) hands-on assistance from their supervisor. This could be addressed in the future by asking the supervisor to validate/confirm the procedure details. Trainees are instructed to register their numbers immediately after completing the procedure to increase the accuracy of the data submitted. If trainees are not consistent about this, the subsequent analysis of total procedures and skills progress will be compromised because the app is synchronized with the smartphone calendar. That said, the lack of gold standard for tracking this important training data is precisely what inspired the development of this app. In summary, we have developed an app, Endostation, that has been successful in collecting meaningful data submitted by endoscopy trainees from a variety of endoscopic procedures. Furthermore, Endostation was able to document and show significant trainee endoscopic skill improvement over time via endoscopic milestones. Further studies are needed to evaluate trainees compliance and comparison with other recording or documentation systems in Gastroenterology.

## Supplementary Information

**Additional file 1:** **Appendix 1.** Menu of Indications for different types of procedures. **Appendix 2.** Menu of therapeutic interventions for different types of procedures. **Appendix 3.** Proposed ASGE grading system for complexity of ERCP procedures [1].

## Data Availability

Project name: EndoStation | Skills Tracker. Copyright: University of Alberta. Project home page: https://endostation.ca Operating system(s): Platform independent. Programming language: PHP/Cordova/MySQL. Other requirements: Modern web browser, Android 8 + , iOS 11 + Any restrictions to use by non-academics: No restrictions. The datasets generated and/or analysed during the current study are not publicly available but are available from the corresponding author on reasonable request.

## References

[1] Cotton PB, Eisen G, Romagnuolo J (2011). Grading the complexity of endoscopic procedures: results of an ASGE working party. Gastrointest Endosc.

[2] Dube C, Rostom A (2016). Acquiring and maintaining competency in gastrointestinal endoscopy. Best Pract Res Clin Gastroenterol.

[3] ASGE Quality Assurance in Endoscopy Committee (2015). Gastrointest Endosc.

[4] Feurer M, Draganov P (2016). Training for advanced endoscopic procedures. Best Pract Res Clin Gastroenterol.

[5] De Witt J, Faulx A, Lightdale J, ASGE Standards of Practice Committee (2017). Guidelines for privileging, credentialing and proctoring to perform GI endoscopy. Gastrointest Endosc.

[6] ASGE Quality Assurance in Endoscopy Committee (2015). Quality indicators common to all GI endoscopic procedures. Gastrointest Endosc.

[7] Prgomet M, Georgiou A, Westbrook JI (2009). The impact of mobile handheld technology on hospital physicians’ work practices and patient care: a systematic review. J Am Med Inform Assoc.

[8] Garritty C, El Emam K (2006). Who’s using PDAs? Estimates of PDA use by health care providers: a systematic review of surveys. J Med Internet Res.

[9] Baumgart DC (2005). Personal digital assistants in health care: experienced clinicians in the palm of your hand?. Lancet.

[10] León SA, Fontelo P, Green L (2007). Evidence-based medicine among internal medicine residents in a community hospital program using smart phones. BMC Med Inform Decis Mak.

[11] Kho A, Henderson LE, Dressler DD (2006). Use of handheld computers in medical education. A systematic review. J Gen Inter Med.

[12] Mosa A, Yoo I, Sheets L (2012). A systematic review of healthcare applications for smartphones. BMC Med Inform Decis Mak.

[13] Dala-Ali BM, Lloyd MA, Al-Abed Y (2011). The uses of the iPhone for surgeons. Surgeon.

[14] Forbes N, Mohamed R, Raman M (2016). Learning curve for endoscopy training: Is it all about numbers?. Best Pract ResClin Gastroenterol..

[15] ASGE task Force (2015). Quality indicators for ERCP. Gastrointest Endosc.

[16] Wani S, Han S, Simon V (2019). Setting minimum standards for training in EUS and ERCP: results from a prospective multicenter study evaluating learning curves and competence among advanced endoscopy trainees 2020. Gastrointest Endosc.

[17] Jorgensen JE, Elta GH, Stalburg CM (2013). Do breaks in gastroenterology fellow endoscopy training result in a decrement in competency in colonoscopy?. Gastrointest Endosc.

