# Supplementary material for: A novel smartphone application for the tracking of procedural numbers and trainee experience in gastrointestinal endoscopy
Source: BMC Med Inform Decis Mak. 2023 Mar 31;23:52. doi: 10.1186/s12911-023-02145-z (PMC10064511; doi:10.1186/s12911-023-02145-z)
Supplement: Supplementary file 1 — Additional file 1: Appendix 1. Menu of Indications for different types of procedures. Appendix 2. Menu of therapeutic interventions for different types of procedures. Appendix 3. Proposed ASGE grading system for complexity of ERCP procedures [1]. [file 12911_2023_2145_MOESM1_ESM.docx]

**Appendix 1.**

**Menu of Indications for different types of Procedures**

**Gastroscopy**

1. Dysphagia
2. Odynophagia
3. Abdominal pain
4. GERD
5. Nausea/Vomiting
6. Iron-deficiency anemia
7. Gastrointestinal bleeding
8. Weight loss
9. Dyspepsia
10. Abnormal findings on imaging study
11. History of peptic ulcer
12. Barrett’s surveillance
13. Suspicion of malignancy
14. Esophageal varices surveillance/portal hypertension
15. GAVE follow-up
16. Suspicion of Celiac disease
17. Surveillance Celiac disease
18. Esophageal ESD (endoscopic submucosal dissection)
19. Gastric ESD
20. Other (free text)

**Colonoscopy**

1. Screening for colorectal cancer (no risk factors)
2. FIT positive
3. Personal history of polyps/cancer
4. Change in bowel habits
5. Rectal bleeding
6. Hematochezia
7. Iron-deficiency anemia
8. Polyposis Syndrome
9. Abnormal findings in imaging study
10. Suspicion of colonic stricture
11. History of diverticulitis
12. Suspicion of inflammatory bowel disease
13. Ulcerative colitis surveillance
14. Crohn’s disease surveillance
15. Melena with negative gastroscopy
16. Chronic diarrhea
17. Weight loss work-up
18. FAP (familial adenomatous polyposis) surveillance
19. Colonic stenting
20. Volvulus
21. Other (free text)

**ERCP**

1. Choledocholithiasis
2. Cholangitis
3. Painless jaundice
4. SOD (sphincter of Oddi dysfunction)
5. Biliary leak
6. Benign Biliary stricture
7. Ampullectomy
8. Abnormal findings on imaging study
9. Pancreatic stricture/stone
10. Post-sphincterotomy bleeding
11. Cholangiocarcinoma
12. Pancreas cancer
13. Spyglass procedure
14. Other (free text)

**Balloon-assisted Enteroscopy**

1. Positive finding at VCE
2. Small bowel bleeding
3. Small bowel stricture
4. Abnormal findings on imaging study
5. Suspicion of IBD
6. Failed colonoscopy
7. Peutz-Jegher syndrome
8. Suspicion of small bowel malignancy
9. Chronic diarrhea
10. Crohn’s Disease small bowel evaluation
11. Suspicion of ulcerative jejunitis
12. Other (free text)

**Endoscopic Ultrasound**

1. Rule out choledocholithiasis
2. Evaluation of submucosal mass esophagus
3. Evaluation of submucosal mass stomach
4. Evaluation of submucosal mass duodenum
5. Evaluation of pancreatic mass
6. Staging esophageal cancer
7. Staging ampullary mass
8. Staging rectal cancer
9. Attempted biliary drainage after failed ERCP
10. Abdominal pain in pancreas cancer
11. Rectal EUS
12. Thoracic EUS
13. Other (free text)

**Appendix 2.**

**Menu of Therapeutic Interventions for different types of Procedures**

**Gastroscopy**

1. Esophageal dilation
2. Pyloric dilation
3. Duodenal dilation
4. Duodenal APC (argon plasma coagulation)
5. Esophageal stenting
6. Gastric stenting
7. Duodenal stenting
8. Esophageal banding of varices
9. Esophageal injection
10. Esophageal hemoclips
11. Esophageal EMR
12. Foreign body removal
13. Esophageal polypectomy
14. Gastric polypectomy
15. Duodenal polypectomy
16. Gastric injection
17. Gastric injection of varices
18. Gastric APC/BICAP
19. Gastric banding of GAVE
20. PEG-tube insertion
21. Naso-jejunal tube insertion
22. Hemospray application
23. Duodenal hemoclips
24. POEM
25. Esophageal ESD (endoscopic submucosal dissection)
26. Gastric ESD (endoscopic submucosal dissection)
27. Esophageal dilation in achalasia
28. Gastric hemoclips
29. Duodenal injection
30. Other (free text)

**Colonoscopy**

1. Colonic injection
2. Polypectomy cold snare
3. Polypectomy hot snare
4. Polypectomy EMR (> 2cm)
5. Colonic stenting
6. Colonic hemoclips
7. Colonic BICAP
8. Colonic dilation
9. Colonic APC (argon plasma coagulation)
10. Colonic decompression (volvulus)
11. Colonic ESD (endoscopic submucosal dissection)
12. Other (free text)

**ERCP**

1. Sphincterotomy
2. Pre-cut
3. Stone extraction
4. Sphincteroplasty
5. Biliary plastic stent
6. Biliary dilation of stricture
7. Pancreatic plastic stent
8. Biliary metal stent
9. Mechanical lithotripsy
10. Pancreatic stent
11. Injection at papilla
12. Ampullectomy
13. Lithotripsy (Spyglass)
14. Other (free text)

**EUS**

1. None
2. Station 1 completed (Trans-gastric view)
3. Station 2 completed (Duodenal bulb view)
4. Station 3 completed (Ampullary view)
5. Celiac plexus neurolysis
6. Fine needle aspiration Pancreas mass
7. Fine needle aspiration Pancreas cyst
8. Fine needle biopsy Node
9. Fine needle biopsy submucosal mass
10. Fine needle biopsy other mass
11. Biliary drainage
12. EUS-guided trans-papillary rendezvous technique
13. Pancreatic pseudocyst drainage
14. Gastric varix injection of coils
15. Other (free text)

**Appendix 3.**

**Proposed ASGE grading system for complexity of ERCP procedures [1]**

Types of Procedures

**Grade 1**

Deep cannulation of duct of interest, main papilla, sampling

Biliary stent removal/exchange

**Grade 2**

Biliary stone extraction < 10 mm

Treat biliary leaks

Treat extrahepatic benign and malignant strictures

Place prophylactic pancreatic stents

**Grade 3**

Biliary stone extraction > 10 mm

Minor papilla cannulation in divisum, and therapy

Removal of internally migrated biliary stents

Intraductal imaging, biopsy, FNA

Management of acute or recurrent pancreatitis

Treat pancreatic strictures

Remove pancreatic stones mobile and < 5 mm

Treat hilar tumors

Treat benign biliary strictures, hilum, and above

Manage suspected Sphincter of Oddi dysfunction (with or without manometry)

**Grade 4**

Remove internally migrated pancreatic stents

Intraductal image-guided therapy

Pancreatic stones impacted and/or > 5 mm

Intrahepatic stones

Pseudocyst drainage, necrosectomy

Papillectomy

ERCP after Whipple or Roux-en-Y bariatric surgery

- Increase 1 level (to a maximum of 4) when the patient has had Billroth II gastrectomy

1. Cotton PB, Eisen G, Romagnuolo J, Vargo J, Baron T, Tarnasky P, Schutz S, Jacobson B, Bott C, Petersen B (2011) Grading the complexity of endoscopic procedures: results of an ASGE working party. Gastrointest Endosc 73:868–874
